# Clinical Characteristics and Genetic Analysis of a Family With Birt-Hogg-Dubé Syndrome and Congenital Contractural Arachnodactyly

**DOI:** 10.3389/fgene.2021.768342

**Published:** 2022-01-19

**Authors:** Jiayong Qiu, Yao Lou, Yingwei Zhu, Min Wang, Huifang Peng, Yingying Hao, Hongwei Jiang, Yimin Mao

**Affiliations:** ^1^ Department of Respiratory Medicine, The First Affiliated Hospital, and College of Clinical Medicine of Henan University of Science and Technology, Luoyang, China; ^2^ Department of Endocrinology, The First Affiliated Hospital, and College of Clinical Medicine of Henan University of Science and Technology, Luoyang, China; ^3^ Department of Radiation Oncology, The First Affiliated Hospital, and College of Clinical Medicine of Henan University of Science and Technology, Luoyang, China

**Keywords:** birt-hogg-dubé syndrome, congenital contractual arachnodactyly, FLCN, FBN2, whole exome sequencing

## Abstract

**Background:** Birt-Hogg-Dubé (BHD) syndrome and congenital contractural arachnodactyly (CCA) or Beals-Hecht syndrome are clinically rare autosomal dominant genetic diseases. In this study, we describe an extremely rare family with BHD syndrome and CCA.

**Objective:** To investigate the clinical and genetic characteristics of a family with BHD syndrome and CCA.

**Methods:** We describe the clinical characteristics, family history, and clinical manifestations of the patient’s family members. The patient underwent a blood test, computed tomography (CT) of the chest, color Doppler ultrasound of the abdomen and heart, and digital radiography of the hands. Whole exome sequencing was performed on his family members.

**Results:** Two years ago, the male proband developed chest tightness and shortness of breath that was accompanied by an irritating cough as well as repeated (four times) spontaneous pneumothorax. The chest CT indicated spontaneous pneumothorax on the right side and cyst and bullae in both lungs. He had no kidney tumors or skin lesions. His son had a history of pulmonary bullae and experienced spontaneous pneumothorax twice. The proband, his mother, and his son were all born with a hand deformity. The sequencing results demonstrated that both the proband and his son had heterozygous variations of the folliculin (FLCN) gene c.1015C > T (p. Gln339Ter) and fibrillin-2 (FBN2) gene c.3485G > A (p. Cys1162Tyr), which are associated with BHD syndrome and CCA, respectively.

**Conclusion:** For patients with chest tightness, shortness of breath, recurrent spontaneous pneumothorax, and congenital hand deformity without inducement, genetic testing should be carried out as soon as possible to make a clear diagnosis, which can then guide treatment and genetic counseling.

## Introduction

Birt-Hogg-Dubé (BHD) syndrome is a rare autosomal dominant genetic disease. Folliculin (FLCN), located on chromosome 17p11.2, is its causative gene. Its common features include multiple fibrofolliculoma, bullae (spontaneous pneumothorax), and kidney tumors (Black et al., 2020). Phenotypically, this condition is highly variable. Even in the same family, affected individuals may exhibit any combination of skin, lung, or kidney manifestations of varying severities (Daccord et al., 2020). Regardless, spontaneous pneumothorax is usually the first manifestation of BHD syndrome (Johannesma et al., 2015), maybe the only one (Xing et al., 2017). In FLCN mutation carriers, skin manifestations usually appear in the fourth decade and gradually increase and become more obvious with age (Iwabuchi et al., 2018). Compared to lung cysts/pneumothorax, the renal manifestations of BHD syndrome occur later (Pavlovich et al., 2005).

Congenital contractural arachnodactyly (CCA) or Beals-Hecht syndrome is a rare autosomal dominant connective tissue disease; it is associated with the disease-causing fibrillin-2 (FBN2) gene. The main clinical features of CCA are spider fingers (toes), flexion fingers, major joint contractures, scoliosis, pectus excavatum, and helix shrinkage (Aivaz et al., 2015). In this paper, we report an extremely rare family with BHD syndrome and CCA.

## Materials and Methods

### Clinical Characteristics of the Family

We describe the clinical characteristics, family, and clinical manifestations of the patient’s family members. Family history: the patient’s parents were married but not intermarried, and both were deceased. The father had no history of symptomatic pneumothorax or hand malformations, while the mother had congenital hand malformations and no history of symptomatic pneumothorax. The patient had two brothers (one has three daughters, one has one son and one daughter) and one sister, and none of these people had no history of symptomatic pneumothorax or hand malformations. The second brother had hemiplegia, and the sister died of cerebral infarction at the age of 40. The patient’s unmarried son had congenital hand malformations, a history of bullae, and spontaneous pneumothorax twice in the local hospital. His son’s condition improved after drainage, and he was physically fit. While the patient denied a history of other familial diseases, his family is considered to have a familial hereditary disease, possibly BHD syndrome.

Blood tests, computed tomography (CT) of the chest, color Doppler ultrasound of the abdomen and heart, rheumatism-related indicators, and digital radiography (DR) of the hands were performed on the patient’s family. Blood samples from the patient and his son were sent to the Henan Rare Disease Research Center for genetic sequencing.

The study was approved by the hospital ethics committee (2021-03-B024), and the family members signed informed consent forms.

### Whole Exome Sequencing (WES)

With their full notification and informed consent, 3 ml of peripheral blood from the patient and his son was drawn and sent to a third-party company for WES. First, the DNA was fragmented, and the library prepared. Then, the Agilent V6 probe was used to hybridize and capture the DNA from the entire exome as well as part of the untranslated region. Finally, the high-throughput sequencing platform was used to detect mutations. Pathogenic genes of single-gene genetic diseases that had been identified in the Online Mendelian Inheritance in Man (June 2019) were analyzed. Please refer to the American College of Medical Genetics and Genomics (ACMG) for standards and guidelines on the interpretation of genetic variations and their grades. Separate clinical analyses of different samples were performed, including clinical symptom matching and disease recommendations.

## Results

### A Rare Family's Characteristics

In the past 2 years, a 55-year-old male patient had chest tightness, shortness of breath, and an irritating cough without obvious triggers. He was repeatedly admitted to the hospital for spontaneous pneumothorax (four times in total).

Physical examination: body temperature, heart rate, and blood pressure were stable, except for shortness of breath. No abnormal findings on skin or mucous membranes upon examination. Weak breathing sounds in the right lung and thick breathing sounds in the left lung. No dry or wet rales were heard. Upon percussion, the right lung made drum-like sounds, while the left lung was voiceless. The heart and abdominal examinations found no abnormalities, but the fingers had bilateral deformities and asymmetries, along with incomplete extension. The patient had a history of hypertension for over a month, and his blood pressure was as high as 180/120 mmHg. His blood pressure was lowered via oral administration of amlodipine besylate, valsartan, and bisoprolol fumarate, and it was controllable. The patient had no bad habits of tobacco or alcohol.

Patient’s blood tests: blood routine, liver and kidney function, and rheumatism-related indicators (including antinuclear antibody profile, anti-neutrophil cytoplasmic-related antibodies, rheumatoid factor, and high-sensitivity C-reactive protein) were normal.

**FIGURE 1:**
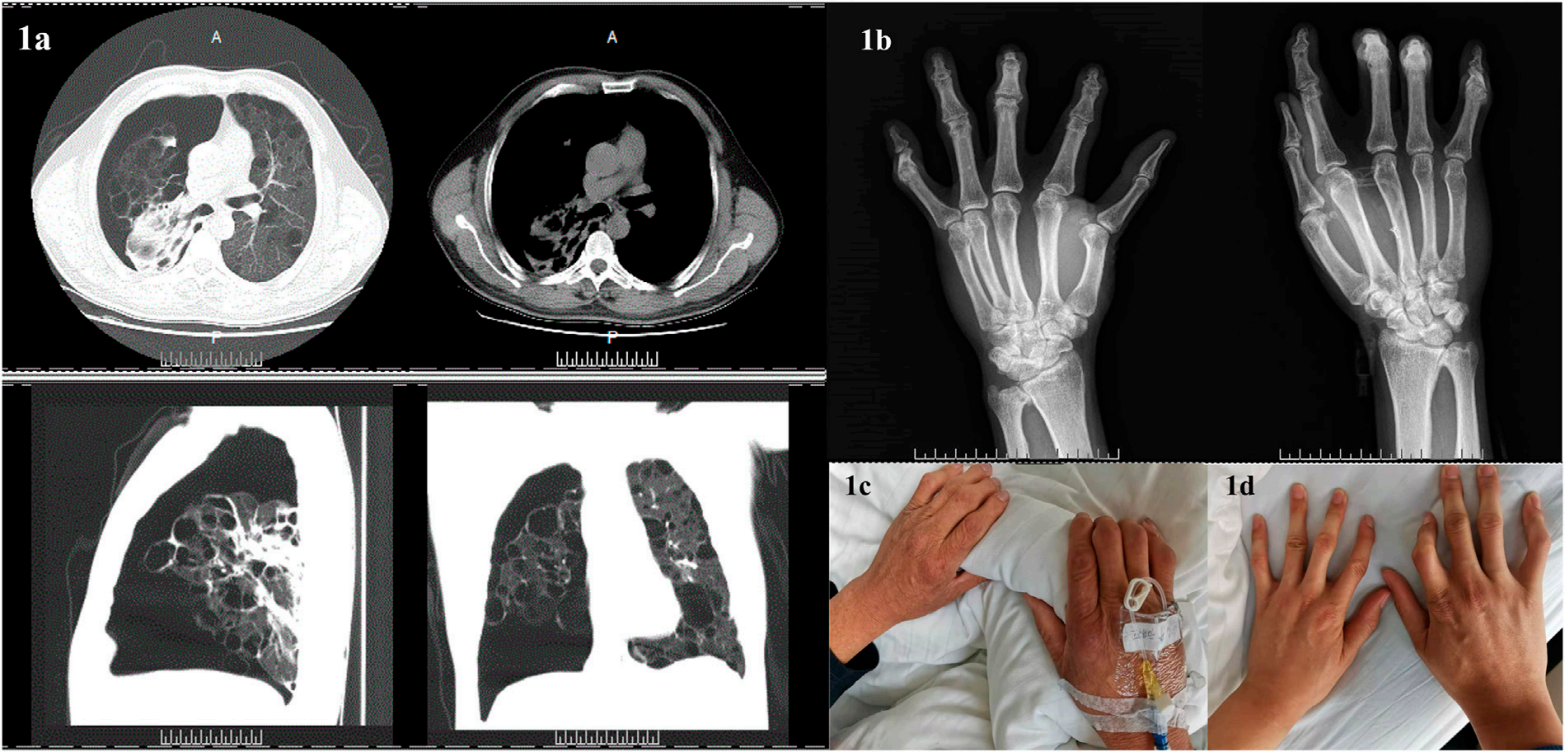
Clinical characteristics of the patient and family members. **(A)**: Computed tomography of the patient’s chest indicated spontaneous pneumothorax on the right side and cyst and bullae in both lungs. **(B)**: Digital radiography in both patient’s hands revealed changes in both hands and wrists, suggesting degenerative changes or chronic inflammatory lesions. **(C)**: The patient’s hands. **(D)**: The hands of the patient’s son.

Chest CT revealed: spontaneous pneumothorax on the right side and cyst and bullae in both lungs (Figure 1A). A thoracic surgeon was consulted and asked to provide closed thoracic drainage to promote lung recruitment. The DR in both hands indicated changes in both the hands and wrists, suggesting degenerative changes or chronic inflammatory lesions (Figure 1B). Both the patient and his son had a history of hand deformities and pulmonary bullae formation (see Figures 1C,D, respectively, for photographs of the patient’s and his son’s hands). No abnormalities were found with the abdominal and cardiac color Doppler ultrasounds. No manifestations of kidney tumors or skin lesions. Pulmonary function test was not performed due to pneumothorax and bullae.

Following treatment, part of the patient’s lung re-expanded, the pneumothorax improved, and air bubbles were still visible in the closed thoracic drainage bottle after the chest tube was intermittently clamped. He was transferred to thoracic surgery for thoracoscopic right lung volume reduction, pleural adhesion cauterization, and pleural fixation. His postoperative recovery was good. No spontaneous pneumothorax recurred during regular telephone and Outpatient follow-up.

### Family WES Results

**FIGURE 2:**
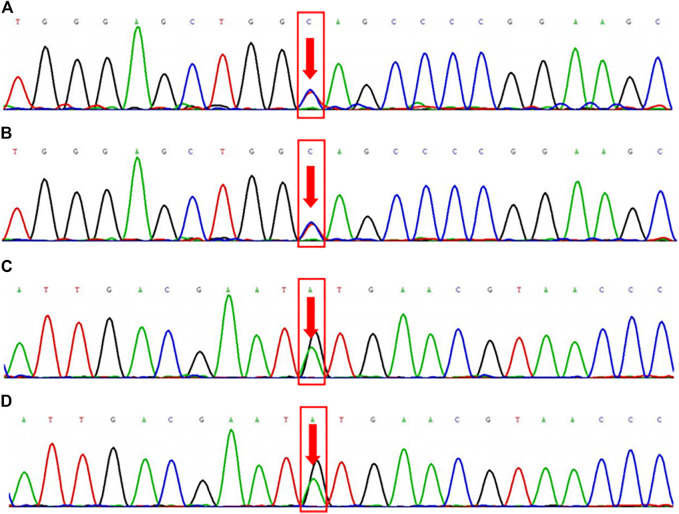
Family whole exome sequencing results. FLCN gene c.1015C > T (p. Gln339Ter) mutation sequencing results in the proband (2a, heterozygous mutation) and his son (2b, heterozygous mutation). FBN2 gene c.3485G > A (p. Cys1162Tyr) mutation sequencing results in the proband (2c, heterozygous mutation) and his son (2d, heterozygous mutation).

The WES results revealed that the proband and the son had heterozygous variants of the FLCN gene c.1015C > T (p. Gln339Ter) and FBN2 gene c.3485G > A (p. Cys1162Tyr) (Figure 2). A heterozygous nonsense mutation was detected in the exon region of the FLCN gene c.1015C > T, resulting in an amino acid change: p. Gln339Ter. The mutation site is reported as a pathogenic variant in the Human Gene Mutation Database (HGMD) but is not included in the ClinVar database. Using the ACMG guidelines, the variant was judged to be pathogenic (PVS1 + PM2 + PP5). A heterozygous missense mutation was also found in the exon region of the FBN2 gene c.3485G > A, resulting in an amino acid change: p. Cys1162Tyr. This mutation is not included in either the HGMD Professional database or the ClinVar database. However, according to the ACMG guidelines, the variant was judged to be potentially pathogenic (PM1 + PM2 + PM5 + PP3).

**FIGURE 3:**
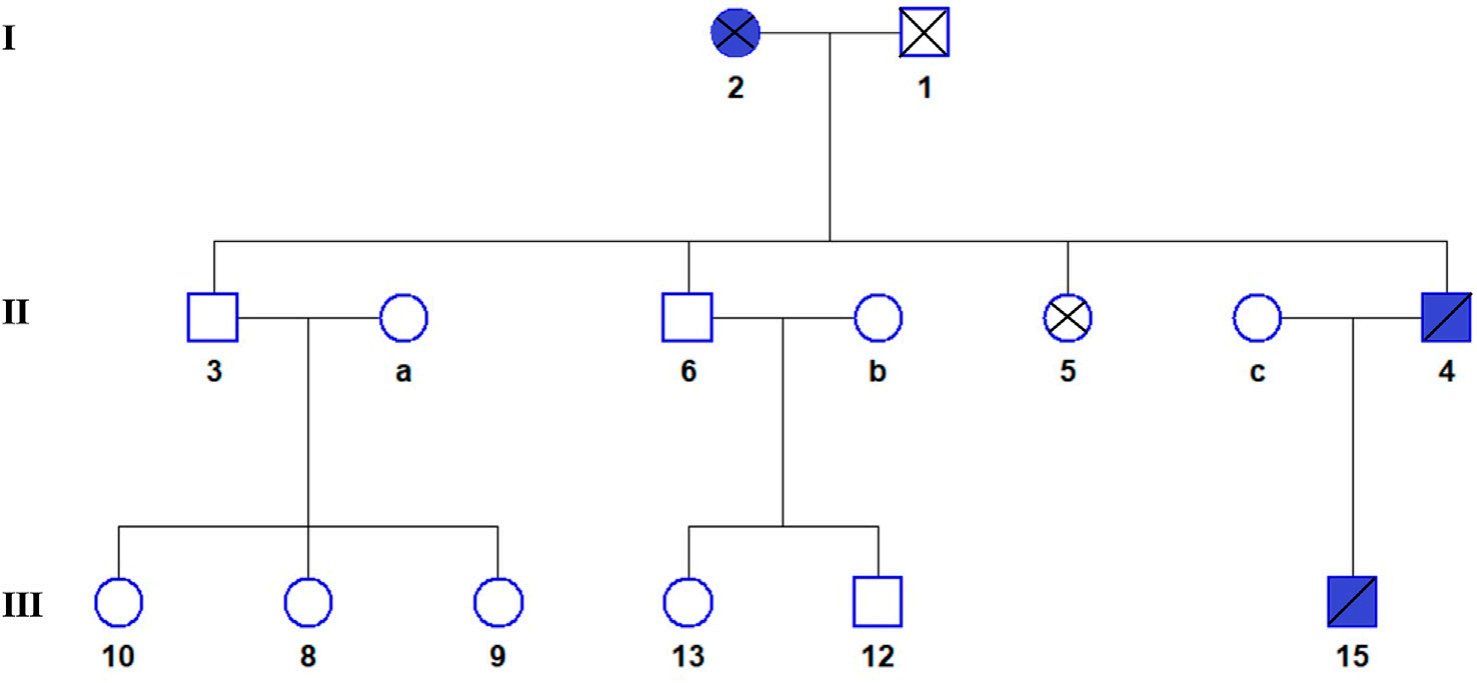
The genealogical tree of Birt-Hogg-Dubé syndrome and congenital contractural arachnodactyly. Blue indicates the patient. The proband (4), his son (15), and his mother (2) all have BHDS and CCA.

The FLCN-gene-associated disease is BHD syndrome, an autosomal dominant genetic disease. The proband and son are consistent with the phenotype of this disease. The FBN2 gene-associated disease is CCA, an autosomal dominant genetic disease. The proband, mother, and son are phenotypically consistent with this disease (see Figure 3 for the genealogical tree). Consequently, the final diagnosis was BHD syndrome and CCA.

## Discussion

In this study, we report for the first time an extremely rare family with BHD syndrome and CCA. This combination has not been reported in China or abroad until now. Compared to Caucasians, Asians with BHD syndrome have a lower incidence of skin and kidney manifestations but a higher rate of pneumothorax recurrence (Park et al., 2017). Indeed, the risk of pneumothorax in BHD patients is 50 times higher than the general population (Kennedy et al., 2016). Pulmonary cysts, multiple and bilateral, occur in 80–100% of patients with BHD syndrome, and 76% of them have pneumothorax. BHD syndrome is one of the most common causes of familial spontaneous pneumothorax (Liu et al., 2020). Thus, a family history of pneumothorax is an important clue that suggests a BHD diagnosis (Gupta et al., 2016). The pulmonary manifestations of BHD syndrome need to be distinguished from other diffuse cystic lung diseases, such as lymphangioleiomyomatosis, Langerhans cell histiocytosis, and lymphocytic interstitial pneumonia (Cui et al., 2016). Unlike other cystic lung diseases, BHD disease does not cause progressive lung function loss or chronic respiratory insufficiency (Liu et al., 2020). According to the literature, the prevalence of renal involvement in BHD patients ranges from 6.5 to 34% (Kunogi et al., 2010). Furuya et al. (Furuya et al., 2016) found that 25.8% of BHD patients have renal damage, especially renal cell carcinoma, the most common histology in chromophobe renal cell carcinoma (43.6%), and all patients with renal involvement also have lung cysts. Kidney cancer is the most serious manifestation of BHD syndrome.

The skin manifestations of BHD syndrome include fibrofolliculoma, trichodiscoma, and perifollicular fibroma. These three types are papules that are between 2 and 4 mm, flesh-colored or light gray-white, and smooth dome-shaped. They are commonly found on the face, neck, and trunk (Aivaz et al., 2015). The most common skin manifestation of BHD syndrome is fibrofolliculoma, where their numbers can range from two to more than 100. Fibrofolliculoma is rare and unique to BHD syndrome and can be diagnosed using needle biopsies (Tong et al., 2018). This patient’s family currently has no skin or kidney manifestations, but they require regular review and follow-ups.

The diagnosis of BHD disease needs to be combined with family history and clinical and/or skin histopathological criteria. Its management mainly includes early pleurodesis in the case of pneumothorax, regular kidney imaging for tumor detection, and diagnostic tests to find BHD syndrome among the patient’s relatives (Gupta et al., 2016). For patients diagnosed with BHD syndrome, follow-ups should be initiated, with special attention to the condition of the kidneys. Currently, pneumothorax is usually treated symptomatically, and electrocoagulation, laser, and curettage are generally used for treating skin lesions.

In this family, both the proband and his son had recurrent spontaneous pneumothorax. First, we must be alert to the possibility of familial pneumothorax. After a clear diagnosis, kidney tumors and skin lesions should be ruled out. If corresponding symptoms occur, seek medical attention in time. It is recommended to screen the genetic locus in members of the subject’s blood-related family, establish a follow-up plan for carriers as soon as possible, and conduct genetic counseling in situations involving fertility. At present, this disease has no special treatment and should be tracked via regular follow-ups.

The main clinical features of CCA are spider finger (toe), flexion finger, major joint contracture, scoliosis, pectus excavatum, and helix shrinkage (Xu et al., 2020). Marfan syndrome (MFS) is a rare autosomal dominant multi-system disease, and it is manifested via bone, eye, skin, and cardiovascular symptoms (Verstraeten et al., 2016). CCA and MFS have many common clinical features, including the so-called Marfan-like appearance, which consists of a tall, slender, and weak appearance as well as skeletal features that include spider fingers, bipedal deformities, pectus excavatum, and kyphosis (Inbar-Feigenberg et al., 2014). However, most patients with CCA have helix shrinkage, flexion contracture, and muscle hypoplasia (Lavillaureix et al., 2017). MFS and CCA are two similar syndromes that are caused by mutations in genes FBN1 and FBN2, respectively (Frédéric et al., 2009). They are difficult to distinguish based on clinical symptoms alone (Gupta et al., 2004); the best way to differentiate them is via genetic testing.

The clinical manifestations of CCA patients are different, involving the heart, bones, lens, and other parts, requiring individualized treatment for the patients. Flexion contractures of the large joints of the extremities often do not require targeted treatment, while hand joint contractures can loosen joints and skin grafts to improve appearance and function. Kyphosis and scoliosis deformity can be corrected by surgery if it affects life. Severe heart deformities often require early surgical treatment, and regular follow-up monitoring is required for non-severe ones (Yin et al., 2020). The proband, mother, and son in this family all have congenital hand deformities with mild symptoms and do not affect normal functions. Since the mother of the proband also has hand deformities, and because the mother is deceased, it is recommended to send samples from the maternal relatives of the proband for screening at this site and establish a follow-up plan for carriers as soon as possible. Genetic counseling when there is a need for fertility.

## Conclusion

WES is currently the gold standard for diagnosing these two familial genetic diseases. For patients with chest tightness, shortness of breath, recurrent spontaneous pneumothorax, and congenital hand deformity without inducement, genetic sequencing should be carried out as soon as possible to make a clear diagnosis, which can guide treatment and genetic counseling. Lifelong follow-up after the diagnosis is made to control the patient’s progress in time and reduce complications.

### Declarations

**Ethical Approval and Consent to participate** The study was approved by the hospital ethics committee (2021-03-B024), and the family members signed informed consent forms.

**Consent for publication** Subject agrees.

**Availability of data and materials** None.

**Competing interests** None.

**Funding** Natural Science Foundation of Henan Province (182300410365).

Science and Technology Project of Henan Province (202102310047).

Medical science and technology Project of Henan Province (2018020285).

The scientific and technological achievements transfer and transformation project of Henan sub-center of SCA (2018105).

**Authors’ contributions** Yimin Mao and Hongwei Jiang designed the study, performed the research. Jiayong Qiu and Yao Lou analysed data and wrote the paper. Huifang Peng collected the data. All authors discussed the results and revised the manuscript.

## Data Availability

The data analyzed in this study is subject to the following licenses/restrictions: none. Requests to access these datasets should be directed to jiayong5201@163.com.

## Abbreviations

ACMG, American College of Medical Genetics and Genomics; ANCA, Anti-Neutrophil Cytoplasmic Antibodies; BHD syndrome, Birt-Hogg-Dubé Syndrome; BHS, Beals Hecht Syndrome; CCA, Congenital contractural arachnodactyly; CT, Computed Tomography; DR, Digital Radiography; FLCN, Folliculin; FBN2, Fibrillin two gene; HGMD, The Human Gene Mutation Database; MFS, Marfan syndrome; OMIM, Online Mendelian Inheritance in Man; UTR, Untranslated Region; WES, Whole Exome Sequencing.
